# Supplementary material for: MET Oncogene Controls Invasive Growth by Coupling with NMDA Receptor
Source: Cancers (Basel). 2022 Sep 11;14(18):4408. doi: 10.3390/cancers14184408 (PMC9496780; doi:10.3390/cancers14184408)
Supplement: Supplementary file 1 [file cancers-14-04408-s001.zip › cancers-1897976-supplementary.pdf]

Table S1. Legend of abbreviations for cancer subtypes classified in “The Cancer Genome Atlas” (TCGA)

Abbreviations	Cancer Name
BLCA	Bladder Urothelial Carcinoma
BRCA	Breast invasive carcinoma
CESC	Cervical squamous cell carcinoma and endocervical adenocarcinoma
COAD	Colon adenocarcinoma
ESCA	Esophageal carcinoma
GBM	Glioblastoma multiforme
HNSC	Head and Neck squamous cell carcinoma
KICH	Kidney Chromophobe
KIRC	Kidney renal clear cell carcinoma
KIRP	Kidney renal papillary cell carcinoma
LIHC	Liver hepatocellular carcinoma
LUAD	Lung adenocarcinoma
LUSC	Lung squamous cell carcinoma
PAAD	Pancreatic adenocarcinoma
PRAD	Prostate adenocarcinoma
READ	Rectum adenocarcinoma
SARC	Sarcoma
SKCM	Skin Cutaneous Melanoma
STAD	Stomach adenocarcinoma
THCA	Thyroid carcinoma
UCEC	Uterine Corpus Endometrial Carcinoma

Table S2. List of primary antibodies used throughout the study

Primary Antibody	Company	Product N.	Experiment
NMDAR1	Santa Cruz	SC-518053	WB
NMDAR2B	Abcam	ab65783	WB/IF
P-NMDAR2B (Tyr1252)	Invitrogen	48-5200	WB/IF
P-NMDAR2B (Tyr1474)	Invitrogen	PA5-37591	WB
MET	Cell Signaling	D1C2 - 8198	WB
P-MET (Tyr1234/1235)	Cell Signaling	3077	WB
Tubulin	SigmaAldrich	051M4771	WB
NMDAR2B	Invitrogen	PA3-105	IHC
MET	R&D	AF276	IHC/IF

Table S3. List of breast cancer cells evaluated for NMDAR1, NMDAR2B, and MET protein expression

CELL LINE	ER	PR	HER2	Subtype	Tumor
**BT-549**	-	-	-	TN	IDC
**MDA-MB-231**	-	-	-	TN	AC
**BT-474**	+	+	+	LB	IDC
**EFM-192A**	+	+	+	LB	AC
**MDA-MB-453**	-	-	+	H	AC
**SK-BR-3**	-	-	+	H	AC
**ZR-75-1**	+	+/-	-	LA	IDC

AC: adenocarcinoma; ER: estrogen receptor; HER2: human epidermal growth factor receptor 2; H: HER2+; LB: luminal B; LA: luminal A; PR: progesterone receptor; TN: triple negative; IDC: invasive carcinoma.

File S1: Western Blot Supplementary Data:
Images and caption of original whole and uncropped western blots

FIGURE 1d

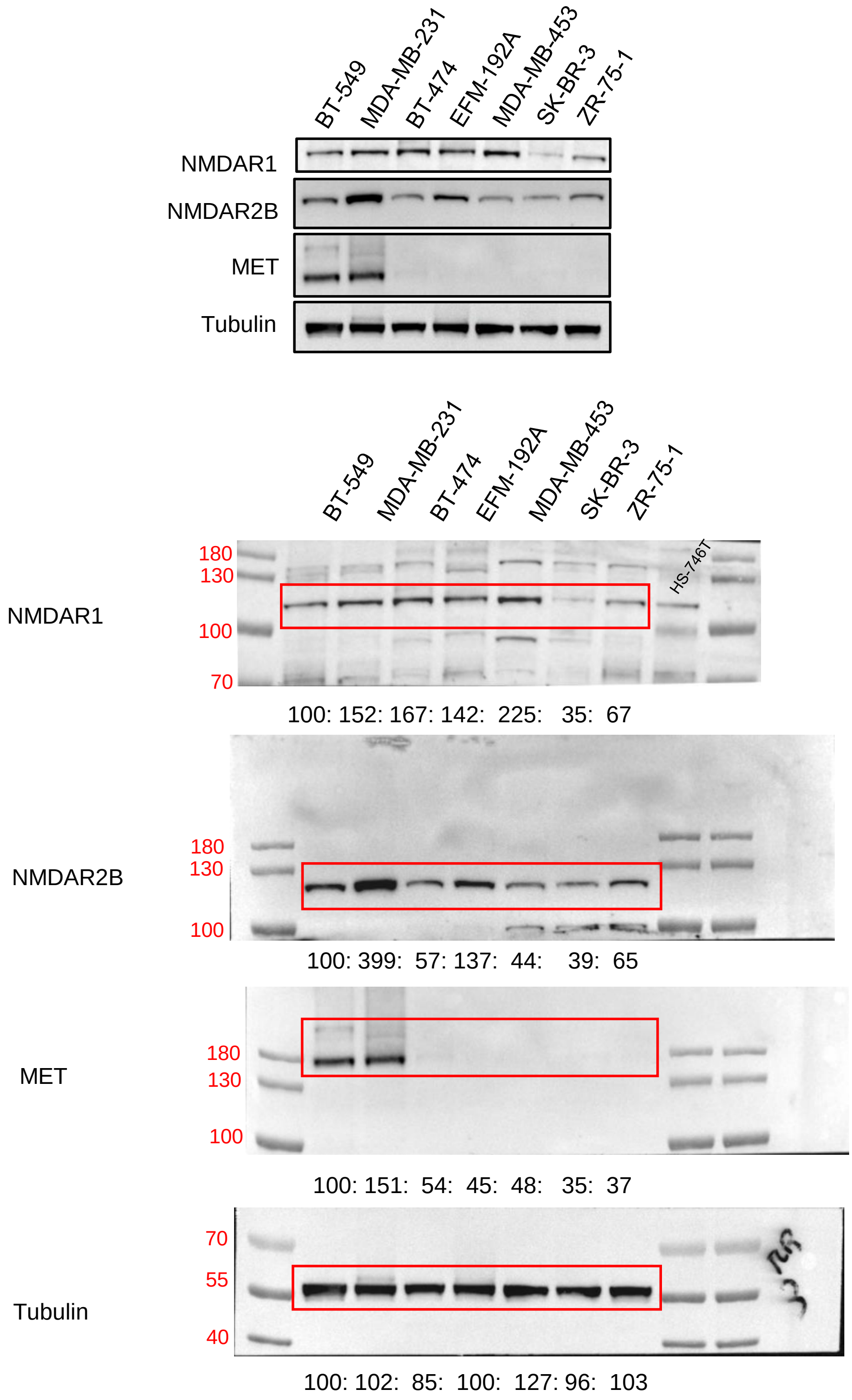

FIGURE 2a

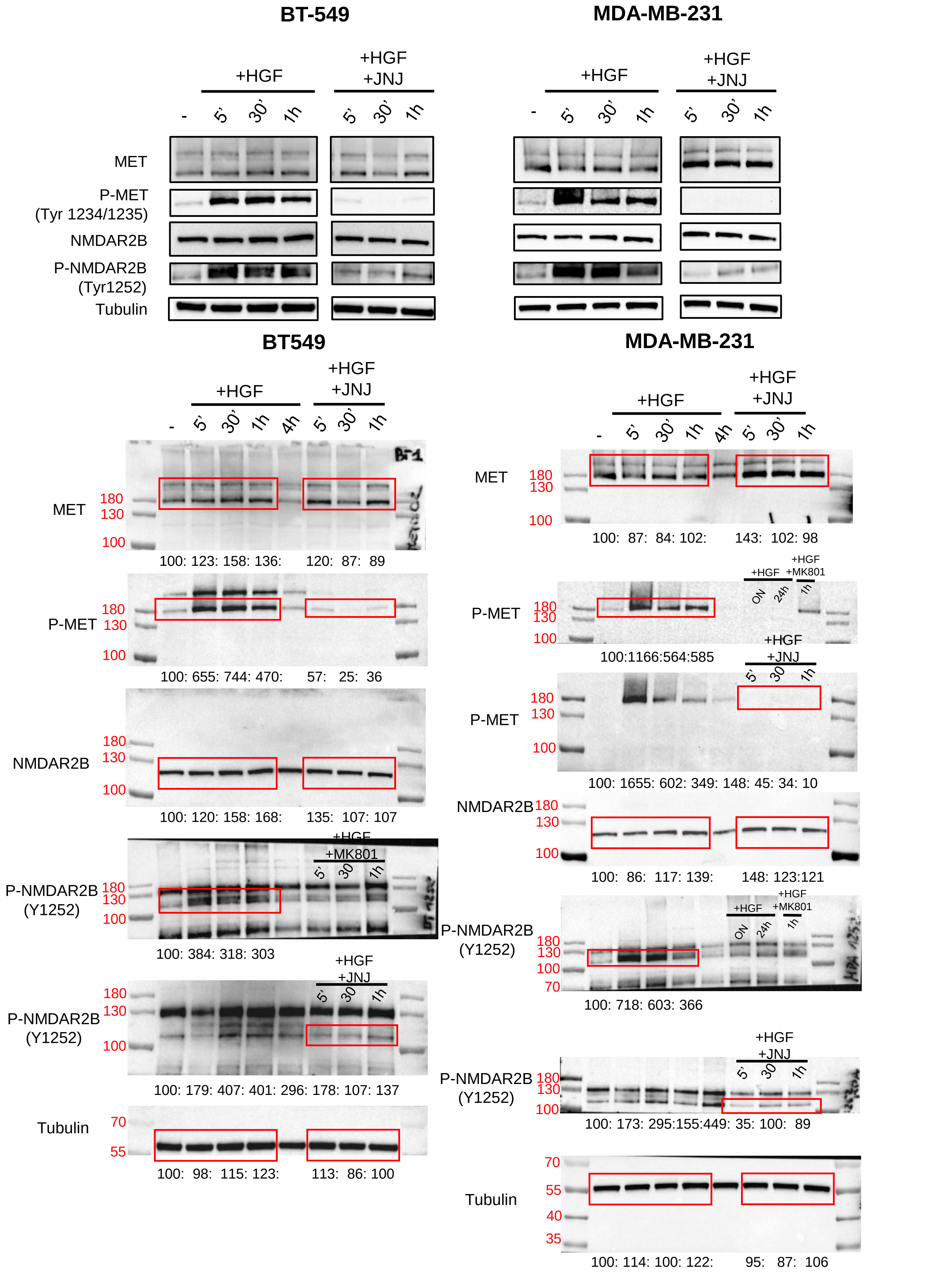
BT-549
MDA-MB-231
+HGF
+HGF +JNJ
- 5' 30' 1h 5' 30' 1h
MET
P-MET (Tyr 1234/1235)
NMDAR2B
P-NMDAR2B (Tyr1252)
Tubulin
BT549
MDA-MB-231
+HGF
+HGF +JNJ
- 5' 30' 1h 4h 5' 30' 1h
MET
180
130
100
100: 123: 158: 136: 120: 87: 89
100: 87: 84: 102: 143: 102: 98
P-MET
100: 655: 744: 470: 57: 25: 36
+HGF
+HGF +MK801
ON 24h 1h
100:1166:564:585
100: 1655: 602: 349: 148: 45: 34: 10
NMDAR2B
100: 120: 158: 168: 135: 107: 107
100: 86: 117: 139: 148: 123:121
P-NMDAR2B (Y1252)
100: 384: 318: 303
100: 718: 603: 366
100: 179: 407: 401: 296: 178: 107: 137
100: 173: 295:155:449: 35: 100: 89
Tubulin
70
55
40
35
100: 98: 115: 123: 113: 86: 100
100: 114: 100: 122: 95: 87: 106

FIGURE 3a

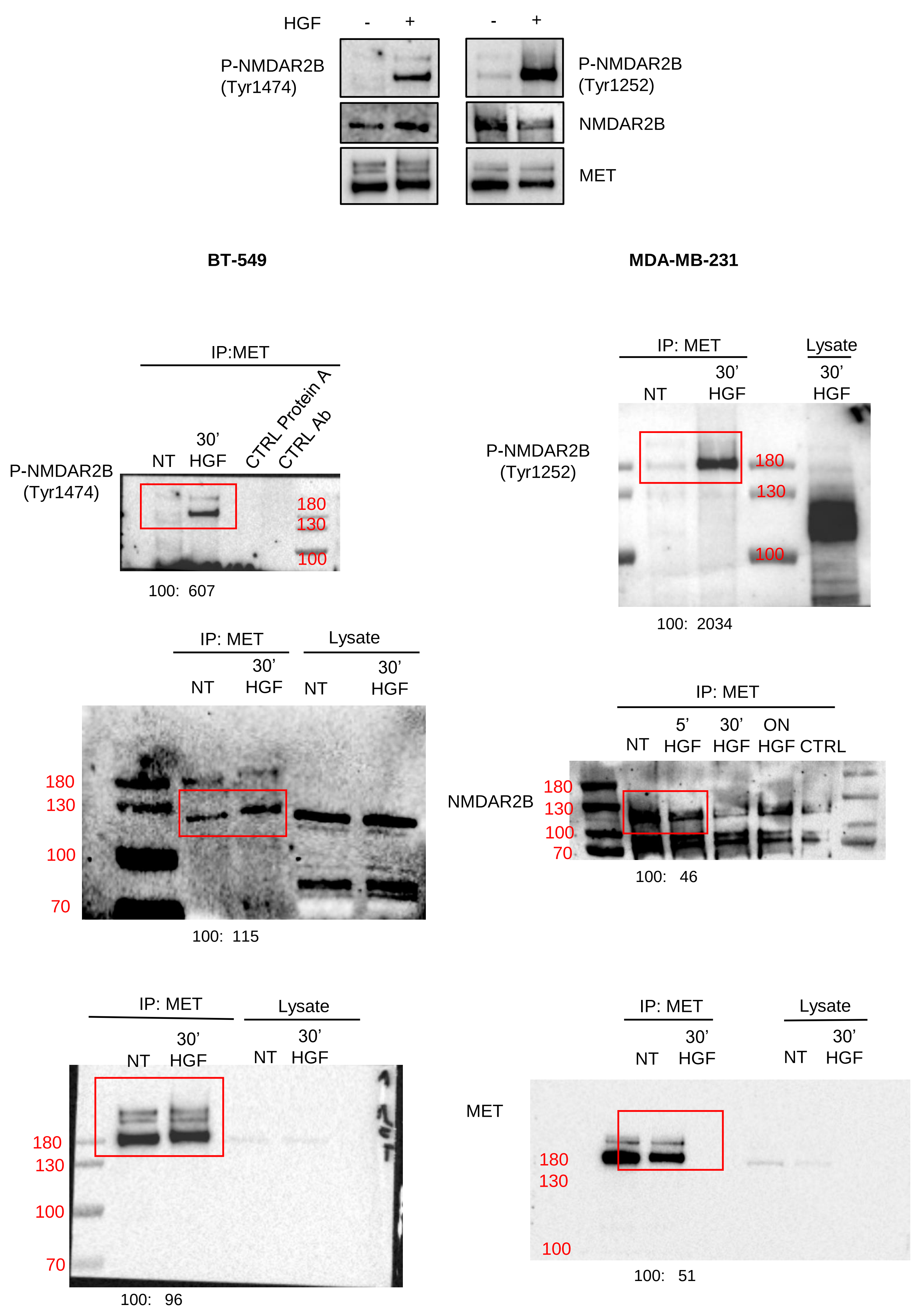
IP: MET
BT-549
MDA-MB-231
HGF
- +
- +
P-NMDAR2B (Tyr1474)
P-NMDAR2B (Tyr1252)
NMDAR2B
MET
BT-549
MDA-MB-231
IP:MET
NT
30' HGF
CTRL Protein A
CTRL Ab
P-NMDAR2B (Tyr1474)
180
130
100
100: 607
IP: MET
Lysate
NT
30' HGF
30' HGF
P-NMDAR2B (Tyr1252)
180
130
100
100: 2034
IP: MET
Lysate
NT
30' HGF
NT
30' HGF
180
130
100
70
100: 115
IP: MET
NT
5' HGF
30' HGF
ON HGF
CTRL
NMDAR2B
180
130
100
70
100: 46
IP: MET
Lysate
NT
30' HGF
NT
30' HGF
180
130
100
70
100: 96
IP: MET
Lysate
NT
30' HGF
NT
30' HGF
MET
180
130
100
100: 51
